# Early stage dental caries detection using near infrared spatial frequency domain imaging

**DOI:** 10.1038/s41598-021-81872-7

**Published:** 2021-01-28

**Authors:** Alistair D. Bounds, John M. Girkin

**Affiliations:** Centre for Advanced Instrumentation, Department of Physics, Durham University, Durham, DH1 3LE UK

**Keywords:** Biophotonics, Imaging and sensing, Dental caries, Optical imaging

## Abstract

Early stage dental caries can be remineralized without the need for “drill-and-fill” treatments that are more invasive and less permanent. However, early stage caries lesions typically present as a white spot on a white background, resulting in many lesions only being identified after they have developed beyond the point of remineralization as cavities. We present a spatial frequency domain imaging technique to characterize the optical properties of dental tissue. This technique enables different dental tissue types (healthy enamel, healthy dentin and damaged or demineralized enamel) to be easily distinguished from one another and allows quantification of the reduced scattering coefficients of dental tissue. The use of near-infrared light at 850 nm allows high depth penetration into the tissue and suppression of absorption effects, ensuring only changes in the reduced scattering coefficient that result directly from demineralization of enamel are observed and simplifying the analysis method. This technique provides a tool to both guide the attention of dentists to areas of interest and potential demineralization, and to provide longitudinal quantified assessments to monitor caries lesion behaviour over time.

## Introduction

Dental caries is the most common chronic disease globally (1st in adults, 6th in children) affecting an estimated 3.9 billion people worldwide. The average yearly expenditure on clinical oral healthcare in Europe between 2008 and 2012 was estimated to be €79bn, which compares with €51bn for cancer, and €55bn for respiratory disease^1^. The early detection of caries, and subsequently less invasive treatment, is clearly an important clinical requirement.

The human tooth consists of an outer layer of enamel, softer dentin and soft tissue (dental pulp) in the pulp cavity, which encloses the dental nerve and supplies blood^2^. The enamel has a crystallite structure, resulting in low optical scattering coefficients at near infrared wavelengths^3^; dentin has significantly higher levels of scattering, but both demonstrate a level of light guiding contributing to low scattering coefficients^4,5^. In a healthy tooth the white color is primarily due to light that is transmitted through the enamel and back-scattered by the dentin. Absorption is low at most wavelengths, and negligible at near-infrared wavelengths^6^.

The path of light is disturbed by the presence of dental caries, resulting in a strong increase in optical scattering at the site of dental caries. However, this does not necessarily make the caries obvious, as caries may present as a white spot on a white background. During a visual inspection a dentist will frequently use air to help any fluid in an early lesion evaporate, leading to an increase in scattering helping to detect early lesions. These early lesions are typically not visible on an X-ray and thus it is generally accepted that at the very early stage dentists currently only detect around 30% of lesions^7^. Thus, light is currently used by clinicians to detect caries but help is required beyond just simple inspection, even after thorough cleaning and drying of the tooth^8^.

At the stage that caries are visible on an X-ray, generally the treatment is the replacement of damaged enamel and dentin with a composite or amalgam material (a filling). These fillings deteriorate with time as bacteria enter microscopic gaps between the filling and the tooth, requiring repeated, larger fillings. However, if the caries can be detected at an early stage, the slow loss of mineral can be reversed, for example with high levels of fluoride, and the enamel can reform. This treatment is known as remineralization and results in restoration of the tooth’s natural structure, avoiding the deterioration associated with fillings. The challenges of filling treatments relative to remineralization treatments is reflected in the worldwide drive to reduce the use of “drill and fill” treatments^9^.

**Figure 1:**
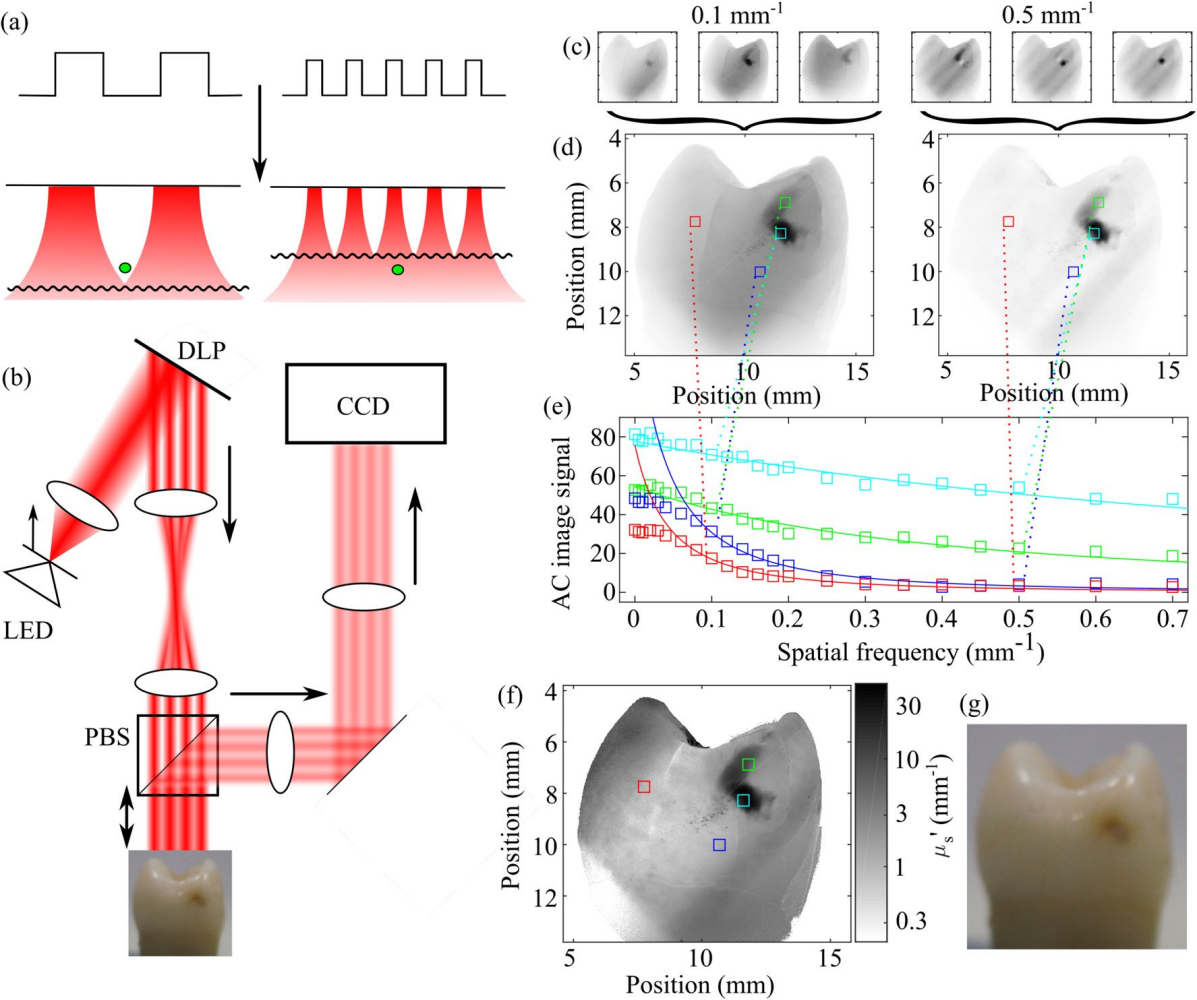
Principles, implementation and analysis techniques in spatial frequency domain imaging. Patterns of light are blurred and attenuated passing through tissue, shown in (**a**). The depth to which the pattern penetrates before being blurred to the point of uniform illumination is illustrated by the curved line. The imaging of a defect (green circle) is phase-dependent at low spatial frequencies (left) but phase-independent at high spatial frequencies (right). (**b**) A collimated LED is encoded with an intensity pattern using a digital light projector (DLP). This pattern of light is transmitted through a telescope and a polarizing beam splitter (PBS) to illuminate an extracted tooth. Specular reflections are transmitted through the PBS, whilst back-scattered light is reflected through an imaging system to a CCD camera. From these images (**c**), the AC image (i.e. the phase-dependent component of the image) can be calculated (**d**). The AC image signal dependence on pattern spatial frequency (**e**) yields the reduced scattering coefficient (**f**), which provides insight not available when using purely visible imaging (**g**).

In the last twenty to thirty years a number of optically based approaches have been developed to detect the early loss of mineral from the tooth^10^. These have ranged from quantifying fluorescence excited by blue light (QLF)^11^ through to infrared thermography^12^ and infrared fluorescence^13^. Direct infrared imaging of teeth in transmission has also been demonstrated as an advance on fiber optics transillumination (FOTI)^14^, though the method described lacks any quantification. Whilst some of these methods are quantitative, they are typically highly sensitive to surface effects such as stains and brown spots^15^, and as a result, none of these methods have become widespread in clinical practice, as demonstrated by the low rate of detection of early-stage dental caries. In the recent past the main optical method being explored for use in the detection of dental caries is polarization enhanced Optical Coherence Tomography^16^ which more recently has been focused on the detection of early white spot caries^17^ where some quantification has taken place. However, these methods are typically slow, as OCT provides point-based detection, and OCT has not reached the level of traction in dental practice that it has in other fields, in particular ophthalmology. These new methods are driven by a widely recognised need to detect dental caries at a sufficiently early stage to treat caries through remineralization. Critically, these techniques must enable detection of caries at a stage when remineralization is an option, and must be quantitative and enable longitudinal tracking. Improved detection without an opportunity for remineralization or assessment and tracking of severity can lead to over-treatment of dental caries that would otherwise naturally remineralize.

Here we present spatial frequency domain imaging (SFDI) as a method to quantify dental optical properties to assess dental caries severity. This is believed to be the first application of SFDI to dental tissue. SFDI is a powerful tool to rapidly and non-invasively assess the optical properties of biological tissue across a wide field of view^18,19^. By illuminating tissue with a pattern of light at multiple spatial phases (illustrated in Fig. 1a with data shown in Fig. 1c) and assessing the phase dependence of the images, the phase-dependent component of the image i.e. an AC (alternating component) image is produced (Fig. 1d). The AC image signal strength has a sensitivity to tissue depth and scattering that is controlled by the spatial frequency of the illuminating pattern^20^. By repeating this at multiple pattern frequencies and assessing the AC image signal dependence on spatial frequency (Fig. 1e), both quantification of the optical properties (Fig. 1f) and depth-profiling of the sample can be achieved^21,22^ to provide more information than available through uniform illumination imaging (Fig. 1g).

A common stumbling block to the clinical translation of SFDI is the mapping of a tissue’s optical properties into a clinically recognizable, or actionable, interpretation. Common techniques to overcome this challenge are to utilize multi- or hyper-spectral techniques^23–25^, enabling assessment of chromophores, for example, to assess oxygenation levels in blood. Such techniques increase the utility of spatial frequency domain imaging, but typically require more advanced systems and longer imaging times.

SFDI, however, is ideally suited to detection and quantitative long-term monitoring of dental caries, as the optical properties of the tissue at a single near-infrared wavelength can be directly related to tissue health. The caries process significantly disrupts the optical properties, and in particular the local scattering, of the dental enamel. SFDI has potential to rapidly image and quantify the scattering properties of the tooth over a wide field of view to directly observe the optical effects of dental caries. Further, as absorption of near-infrared light is negligible^6^ in dental tissue the analysis of data is substantially reduced, allowing near-realtime analysis of SFDI data to be achieved and allowing the technique to solely target biologically relevant measurements. Near infrared light is non-ionising and poses no risk to the patient, meaning that a problem area can be reexamined on a regular basis, the process of remineralization quantified and the treatment protocol modified if required. Further, the use of near infrared light enables the use of low-cost LEDs and silicon detectors.

## Results

### Tissue characterization

**Figure 2:**
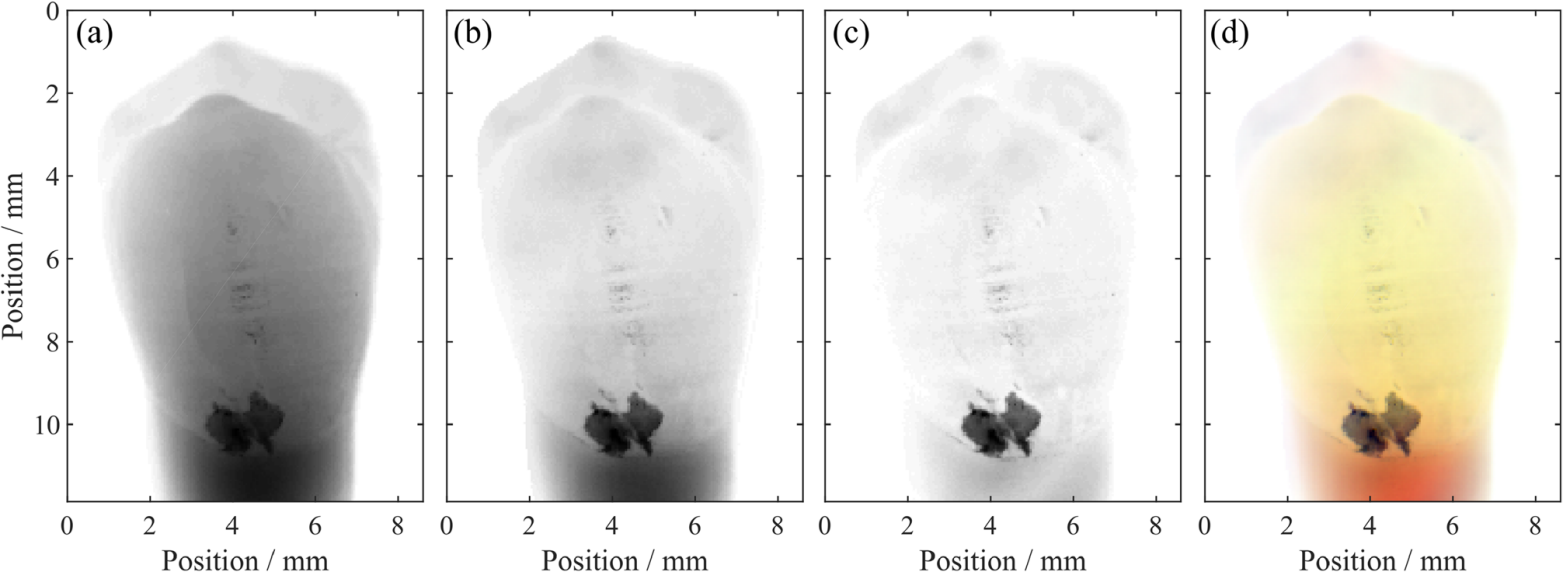
AC images at (**a**) 0.05 $\hbox {mm}^{-1}$, (**b**) 0.2 $\hbox {mm}^{-1}$ and (**c**) 0.5 $\hbox {mm}^{-1}$. (**d**) Combining these three data-sets into a single RGB image allows easy distinction between enamel (yellow), dentin (red) and damaged tissue (black). Damage may be due to caries such as those shown in Fig. 3 or caused during extraction, such as that shown here.

AC images of an extracted human tooth are shown in Fig. 2 at three spatial frequencies.

It is immediately clear that the very different optical properties of enamel, dentin and damaged enamel are readily distinguishable based on the AC images at different spatial frequencies. At low spatial frequencies (0.05 $\hbox {mm}^{-1}$) there is limited difference between all tissue types, the white-spot-on-white-background problem. At intermediate spatial frequencies (0.2 $\hbox {mm}^{-1}$), enamel AC image signal falls significantly, as the low reduced scattering coefficient of healthy enamel results in long photon migration and significant pattern blurring. Dentin and damaged tissue demonstrate sufficient scattering to reduce photon migration and have relatively high AC image signal. At high spatial frequencies (0.5 $\hbox {mm}^{-1}$), only the damaged tissue has sufficiently high scattering to back-scatter light before significant pattern loss has occurred, and thus only damaged tissue has high AC image signal at high spatial frequencies. Normalizing these images to the peak signal and combining the three images in the form of a three-color image (Fig. 2d, we can therefore readily distinguish enamel (yellow), dentin (red) and damaged tissue (black), offering a simple tool to guide the attention of a dentist.

### Quantification of optical properties

**Figure 3:**
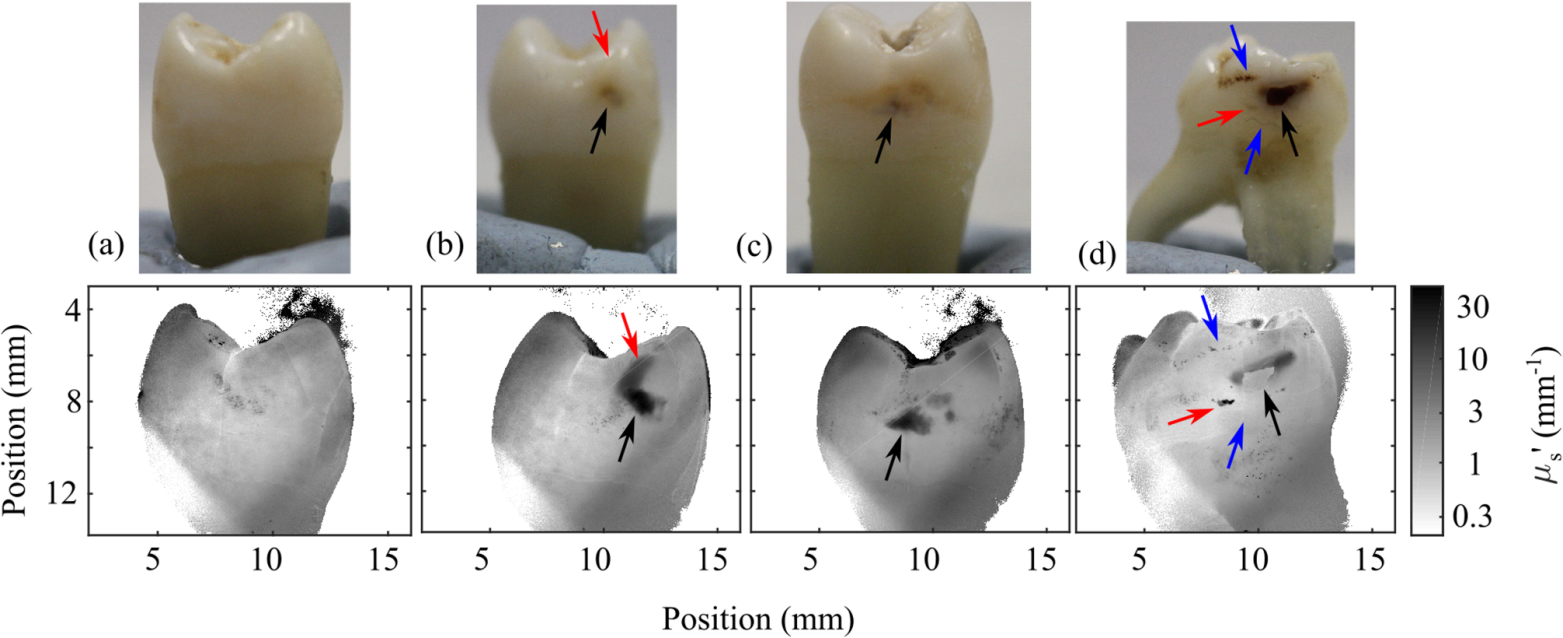
Visible images (top) and reduced scattering coefficients (bottom) for four teeth labelled (**a**–**d**). Black arrows point to cavities identified both with visible imaging and through reduced scattering coefficient, red arrows highlight cavities or demineralization identified only through reduced scattering coefficient, and blue arrows highlight surface defects that are observed in visible images but that do not significantly affect reduced scattering coefficient.

The dependence of AC image signal on spatial frequency is a function of reduced scattering coefficient $\mu ^{'}_{\mathrm {s}}$. By fitting to this function to obtain $\mu ^{'}_{\mathrm {s}}$ (considered further in the Methods section), the high contrast between the optical properties of different regions of tissue is observed in reduced scattering coefficient $\mu ^{'}_{\mathrm {s}}$, shown in Fig. 3 alongside visible images of extracted human teeth.

Caries lesions are readily observed due to their high reduced scattering coefficients. Typical reduced scattering coefficients taken from healthy tissue vary from 0.5 to 1.5 $\hbox {mm}^{-1}$. These values are consistent with literature measurements of scattering coefficients in enamel and dentin once directionality effects to convert between scattering coefficients and reduced scattering coefficients are accounted for^6^. Caries lesions identified through spatial frequency domain imaging have far higher reduced scattering coefficients, reaching values of $\mu ^{'}_{\mathrm {s}}=30$ $\hbox {mm}^{-1}$, which is expected given the damage to the hydroxyapatite rods caused by demineralization.

The lowest spatial frequencies used in this fitting occur when the pattern period is approximately equal to the width of a tooth; at spatial frequencies below this the model does not accurately reflect the boundary conditions imposed by the finite size of the tooth. This effect is particularly significant for healthy tissue with low reduced scattering coefficients; higher photon migration makes discontinuities at the tooth boundary more significant. Very high spatial frequencies have low AC image signals and are thus very sensitive to noise. Consequently, fitting is performed for data taken with spatial frequencies ranging from 0.08 to 0.5 $\hbox {mm}^{-1}$. This range is smaller than the spatial frequency range presented in Fig. 2, as the qualitative nature of the tissue-based distinction shown in Fig. 2 is less sensitive to these effects.

**Figure 4:**
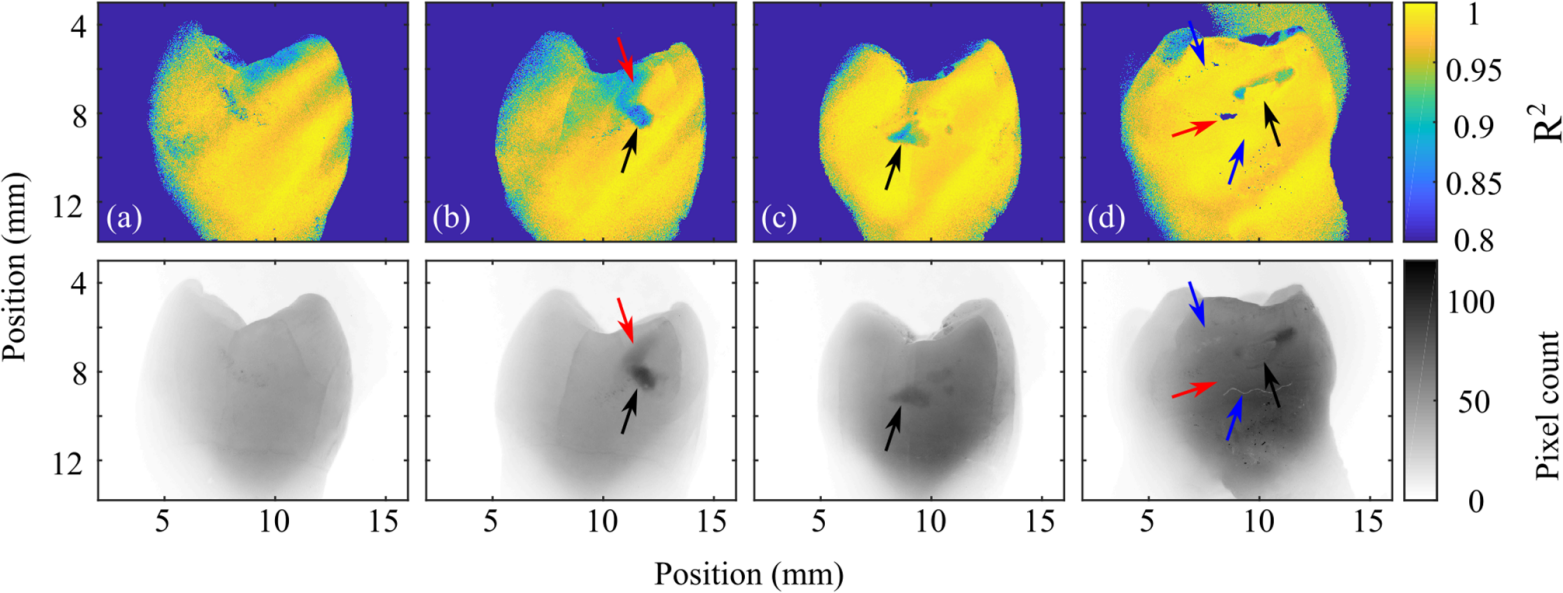
Quality of fit (top) and uniform illumination images (bottom).

Fit quality is assessed using $R^{2}$ fit coefficients shown in Fig. 4 and is generally very good, yielding $R^{2}$ values of 0.97-0.995, offering confidence in our quantification techniques. Dental caries have lower quality fits than healthy tissue. This is attributed to two factors. Firstly, whilst teeth are kept hydrated prior to imaging, they are exposed to air during imaging, causing them to dry. As fluid in porous regions of the tooth is replaced with air, the refractive index mismatch between tooth and environment increases, resulting in greater scattering. This affects damaged tissue more significantly, as it is easier for water to leave the damaged tissue. As imaging initially begins at low spatial frequencies, this effectively increases the observed scattering at high spatial frequencies and is also observed in the DC images. This effect is small, typically changing the DC image signal by less than 1% and is not an issue that will affect in-vivo measurements due to the natural humidity of the mouth and achievable increases in imaging speed.

Secondly, and more fundamentally, the quantification model is based on a homogeneous medium. Dental caries and cavities are not homogeneous, and thus are ill-suited to this fitting model. Future work may improve on this simple model by accounting for a two-layer system. Despite these limitations, fit qualities of $R^{2}\sim 0.85$ are still adequate to demonstrate materially different optical properties of the tissue; simply demonstrating the inaccuracy of the model for these regions is clear evidence of disrupted dental tissue. Absorption at near infrared wavelengths is negligible and therefore is not included in the fitting, although the effects of absorption on the fitting technique are discussed further later.

## Discussion

**Figure 5:**
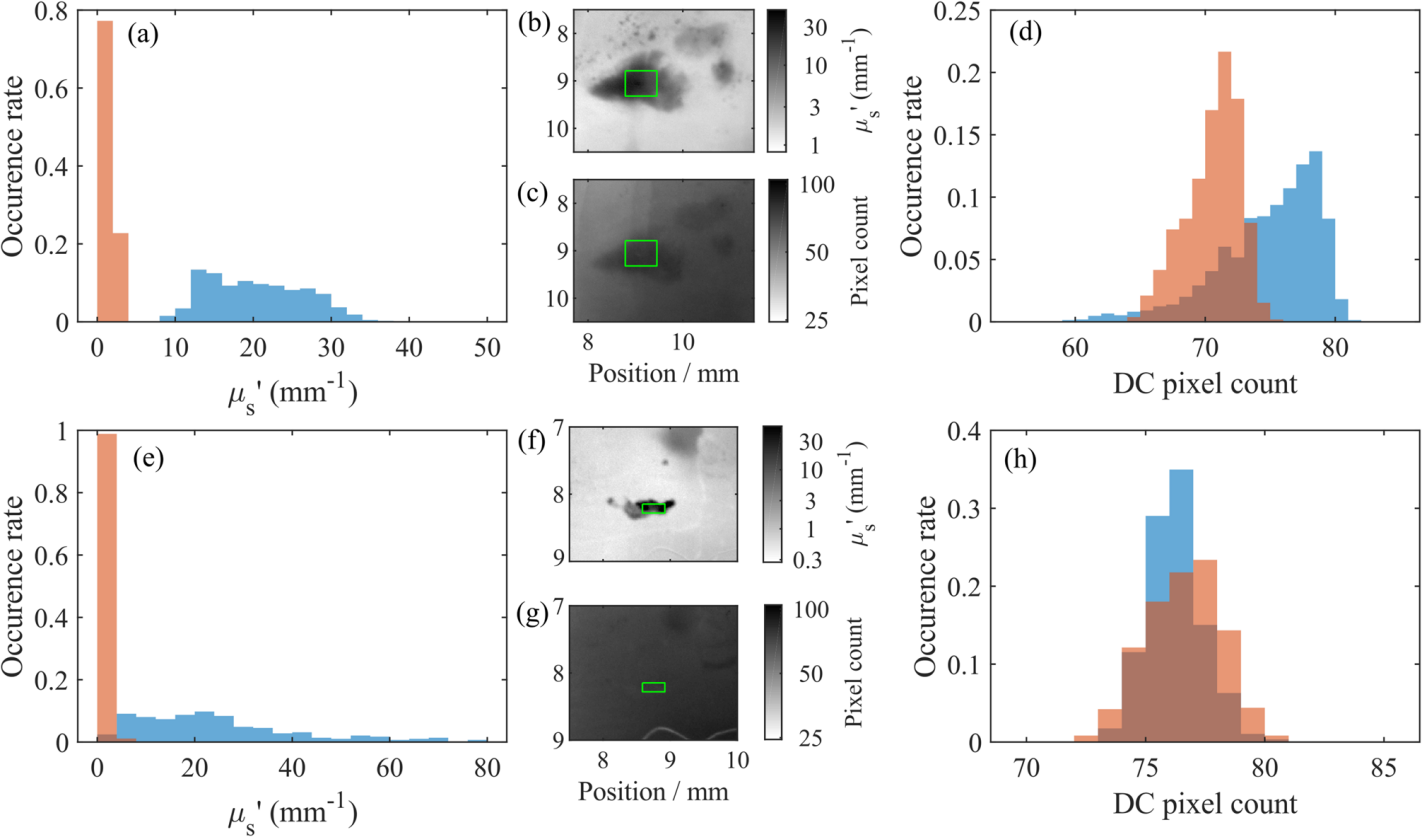
Reduced scattering coefficient (**a**, **e**) and uniform illumination pixel count (**d**, **h**) taken from the tooth in Fig. 3c (top) and d (bottom) for cavities (blue) and healthy tissue (orange). Occurrence rate is the probability of a pixel scattering coefficient being in each bar. The center column shows the cavity reduced scattering coefficient (**b**, **f**) and near infrared uniform illumination (**c**, **g**), both shown on a logarithmic scale. The green box shows the caries shown in the histograms, the healthy tissue shown in the histogram is taken from healthy regions of the same tooth (not shown in the images).

It is clear from Figs. 3 and 4 that assessment of reduced scattering coefficient provides information beyond what visible and near-infrared images alone can show. All caries observed in visible images (highlighted in Fig. 3 with black arrows) are readily and quantifiably observed in reduced scattering coefficient, whilst surface defects (blue arrows) are suppressed in the reduced scattering coefficient as they do not significantly affect subsurface scattering. Two areas of demineralization are identified by red arrows that are easily observed in the scattering coefficient but not through visible imaging alone. The tooth in Figs. 3 and 4d shows a cavity (black arrow) that has resulted in complete loss of material, appearing very striking in the visible spectrum, but as the loss of tissue is complete, the cavity is not detected through the reduced scattering coefficient. Instead, the disruption surrounding the cavity is much more apparent and a small caries lesion slightly below the main cavity can also be observed through the reduced scattering coefficient despite being only very weakly apparent in the visible image. This carious lesion identified by the red arrow in Fig. 3d is shown further in Fig. 5e–h.

Figure 5 shows a detailed, zoomed-in image of the cavities shown in Fig. 3c and d, shown through scattering coefficient and near-infrared uniform illumination. Histograms of reduced scattering coefficient and uniform illumination images illustrate the enhancement in contrast between carious tissue and healthy tissue. In Fig. 5b, the main cavity ($\mu _s^{'}=25$ $\hbox {mm}^{-1}$), two minor cavities ($\mu _s^{'}=6$ $\hbox {mm}^{-1}$) and some further demineralization above the main cavity ($\mu _s^{'}=6$ $\hbox {mm}^{-1}$) are easily distinguished from surrounding tissue ($\mu _s^{'}=1.5$ $\hbox {mm}^{-1}$) due to the change in scattering coefficient, whilst using uniform illumination (Fig. 5c) contrast is very poor, changing only by approximately a maximum of 10%.

The benefits of reduced scattering coefficient measurements using near infrared spatial frequency domain imaging are even more striking in the second case (Fig. 5e–h), where scattering from an early-stage cavity ($\mu _s^{'}=20$ $\hbox {mm}^{-1}$) is a factor of 40 greater than surrounding tissue ($\mu _s^{'}=0.5$ $\hbox {mm}^{-1}$). Despite this difference in reduced scattering coefficient, this cavity is barely visible under uniform illumination, with surface effects being much more visible, such as the hair-line marking at the bottom of the tooth observed in the uniform illumination image.

Spatial frequency domain imaging clearly has the capability to guide the attention of a dentist to highly optically scattering dental tissue that requires consideration by the dentist. The quantifiable nature of this imaging may provide a more powerful dental tool, allowing the monitoring of caries lesion behaviour over time to assess the effectiveness of treatment or changes in condition severity. The level of optical scattering may be correlated with severity of dental caries, thus informing optimum treatment pathways.

Finally, we note the use of co-aligned illumination and imaging axes, which allows this system to be made compact without compromising detection efficiency. Based on the optical intensity requirements and existing camera and DLP technology, it is practical to produce a device that can image a tooth at the 13 spatial frequencies used in the fitting, each at three phases, in approximately 0.5 seconds with optical intensities that are high enough to produce high quality images whilst being well below ocular maximum permissible exposure.

This system is thus highly practical for translation from the laboratory into a clinical setting. Imaging times of less than one second to obtain a wide-field quantitative image of an entire tooth are acceptable to patients and clinical users, and are substantially faster than typical OCT timescales to image a full tooth. A resolution of $13\,\upmu \hbox {m}$ as presented here is easily sufficient to detect the smallest of early-stage dental caries. A full study of the efficacy of this system in detecting early-stage dental caries would require a large-scale trial beyond the scope of this work, and as early-stage dental caries are so easily missed, there is limited data to characterise the expected deviation in scattering coefficient. However, we have demonstrated in Fig. 5 that very substantial deviations in dental caries scattering coefficients can be observed even without associated deviation in uniform illumination signal, offering confidence in the technique’s ability to detect early signatures of dental caries that would otherwise go unnoticed. Further, we also demonstrate in Fig. 3 suppression of surface effects that can inhibit quantitative assessment in other imaging methodologies. The materials required for this imaging system (primarily a camera, a digital mirror device or projector, and a near infrared LED) are low-cost, reliable and widely available. Finally, there is no ionizing radiation present and thus the procedure could be repeated as frequently as is felt to be necessary and there is no limitation in the potential use on children.

### Effect of absorption

SFDI is commonly used to calculate both absorption and reduced scattering coefficients. In this work, absorption is negligible as the enamel absorption lengthscale is comparable to the typical thickness of the enamel layer. We therefore exclude absorption from the fitting model, reducing the number of parameters required to fit drastically accelerating the fitting process, whilst reducing sensitivity to over-fitting.

To verify that this treatment is reasonable, we have repeated the fit whilst allowing for the effects of absorption, following the same fitting method described below. We find that across 80% of the image, the best fit occurs for an absorption coefficient of 0 $\hbox {mm}^{-1}$. Regions in which non-zero absorption is observed typically occur in regions with lower signal and yield average absorption coefficients of 0.15 $\hbox {mm}^{-1}$, well below the observed reduced scattering coefficients.

Whilst absorption coefficients of 0 $\hbox {mm}^{-1}$ are not realistic, this is expected given the manner in which AC image signal varies with spatial frequency, and demonstrates that the level of absorption observed in dental tissue is negligible. The effect of absorption in an AC image signal is only present at low spatial frequencies; the weaker the effect of absorption, the lower the spatial frequency at which effects are present. As absorption of near infrared light is so low (and thus only likely to affect very low spatial frequencies), and as tissue quantification is only conducted at spatial frequencies greater than 0.08 $\hbox {mm}^{-1}$, it is expected that absorption will not play a role in the quantification of tissue optical properties.

This is further verified by consideration of the coefficient of determination ($R^{2}$). Without including absorption effects we calculate average values of $R^{2} \sim 0.97$-0.995, with negligible change ($<0.005$) when including the the effect of absorption. This demonstrates that less than 3% of the variance in AC image signal with spatial frequency is unaccounted for when considering only scattering effects and validating the assumption that the effect of absorption is negligible.

In this work we only consider near infrared light, as this allows us to exclude absorption as described above. This method could readily be adapted to wavelengths at which absorption is more significant, but for the purposes of dental caries, it is sufficient to only characterise the role of scattering and benefit from the improved fitting that results from this.

## Methods

### Experimental set-up

The principles and requirements of SFDI systems are well-established, with details and requirements published elsewhere^26,27^, albeit typically imaging soft tissues (e.g. skin). We therefore offer only an outline of the principles of SFDI and briefly describe the system lay-out, which are illustrated in Fig. 1a and b. The depth to which a pattern of light will penetrate through tissue before being blurred to uniform illumination will depend on both the tissue optical properties and the pattern frequency. Thus, by assessing the phase-dependent and phase-independent components of exposures at different spatial frequencies, depth profiling and tissue quantification can be achieved. For example, the imaging of the defect represented in green in Fig. 1a is phase-dependent when illuminated at low spatial frequencies but not phase-dependent at high spatial frequencies, which are blurred to uniform illumination before reaching the defect due to scattering.

The output of an LED emitting at 850 nm (Thorlabs M850D3) is collimated and relayed to a digital light projector (DLP) (chip: Texas Instruments DMD Discovery 1100, interface: Vialux Accessory Light modulator Package). The pattern encoded onto this illumination light is then relayed to the image plane through a telescope and a polarizing beam splitter (PBS). Light backscattered from the image plane is reflected by the PBS and imaged via a 4-f lens system onto a camera (QImaging QIClick), giving a calculated and measured resolution of $13\,\upmu \hbox {m}$ and a field of view of 13.9 mm by 18.6 mm. Specular reflections from the tooth surface are transmitted by the PBS, ensuring that only back-scattered light is imaged. This approach uses co-aligned illumination and detection axes to maximize detection efficiency compared to designs with small angles between illumination and detection axes; an important consideration when used in the confined space of the oral cavity.

A custom LabVIEW program controls the DLP and the camera. Typical camera exposure times are 50 ms/image, although the camera and control program limit imaging speeds to approximately 2 fps, with illumination intensities of 0.5 mW/$\hbox {cm}^2$. The projection field of view is approximately 1 $\hbox {cm}^2$, less than the imaging field of view, and is rotated to a diamond shape. The pattern of light $I_{{\rm illumination}}$ is a 1D sinusoidal wave with an offset:

1 $$\begin{aligned} I_{{\rm illumination}} (x) = \frac{I_{{\rm max}}}{2} [1 + \sin {(2 \pi f x + \phi )}] \end{aligned}$$

here $I_{\mathrm {max}}$ is the maximum intensity, *f* is the pattern spatial frequency, *x* is the spatial position and $\phi$ is the phase. For each spatial frequency, the tooth is illuminated by the same pattern three times with spatial phases of 0$^{\circ }$, 120$^{\circ }$ and 240$^{\circ }$. Spatial frequencies vary from DC illumination to 1 $\hbox {mm}^{-1}$, although a smaller range of spatial frequencies are used in the analysis.

Imaging is performed on extracted human teeth. All procedures have been approved by the local ethics committee within the Department of Physics, Durham University (PHYS-2020-02-06T16:45:01-vdxs54). Teeth were collected at Dundee Dental School after obtaining written informed consent under the auspices of the Tayside Tissue Bank, NHS Tayside, and were extracted as part of routine procedures and kept in accordance with the UK Human Tissue Act. The teeth are kept hydrated and are mounted in a mounting putty before being imaged with a DSLR camera and the SFDI set-up. A black enclosure minimizes stray light in the SFDI system.

### Demodulation

Each exposure (shown in Fig. 1c for two spatial frequencies and three spatial phases) contains a phase-sensitive component and a phase-insensitive component that is dependent on spatial frequency but not on spatial phase. To separate the phase-sensitive component (referred to as an AC image $I_{\mathrm {AC}}$ and shown in Fig. 1d), from the raw data, at each spatial frequency, the differences between exposures at different spatial phases are added in quadrature^18^:

2 $$\begin{aligned} I_{\mathrm {AC}}(f) = \frac{2^{\frac{1}{2}}}{3} \Big [\big (I_{1}(f,\phi _{1}) - I_{2}(f,\phi _{2})\big )^{2} + \big (I_{2}(f,\phi _{2}) - I_{3}(f,\phi _{3})\big )^{2} + \big (I_{3}(f,\phi _{3}) - I_{1}(f,\phi _{1})\big )^{2}\Big ]^{\frac{1}{2}} \end{aligned}$$

This technique thus ensures that the uniform component that is present in all exposures is removed, leaving only the AC image (shown in Fig. 1d). The first term $\frac{2^{\frac{1}{2}}}{3}$ is a normalization factor, and $I_{1}(f,\phi _{1})$, $I_{2}(f,\phi _{2})$, and $I_{3}(f,\phi _{3})$ represent exposures with the three different spatial phases $\phi$ at the same spatial frequency *f*. A DC image equivalent to uniform illumination can also be produced from the normalized sum of exposures:

3 $$\begin{aligned} I_{\mathrm {DC}} = \frac{1}{3} [I_{1}(f,\phi _{1}) + I_{2}(f,\phi _{2}) + I_{3}(f,\phi _{3})] \end{aligned}$$

### Tissue quantification

Tissue quantification techniques for diffuse imaging are well-developed, including Monte Carlo models^28^, look-up tables^29^ and analytical approaches^30^ based on either the radiative transfer equation (RTE) or the diffusion equation, a simpler approximation to the RTE. We use a pixel-based analytical solution based on the diffusion equation developed by Cuccia et al.^21^ to quantify optical properties from AC images.

4 $$\begin{aligned} I_{{\rm AC}} (f) = \frac{3 A \alpha ^{'}}{\Big (\frac{\mu ^{'}_{{\rm eff}}}{\mu _{{\rm tr}}} + 1\Big )\Big (\frac{\mu ^{'}_{\mathrm {eff}}}{\mu _{\mathrm {tr}}} + 3A\Big )} \end{aligned}$$

The transport coefficient $\mu _{\mathrm {tr}} = \mu _{\mathrm {a}} + \mu ^{'}_{\mathrm {s}}$ characterises the mean free path of photons before absorption or scattering events occur, and is calculated from the absorption coefficient $\mu _{\mathrm {a}}$ and the reduced scattering coefficient $\mu ^{'}_{\mathrm {s}}$. The reduced scattering coefficient $\mu ^{'}_{\mathrm {s}}= \mu _{\mathrm {s}} (1-g)$ comes from the scattering coefficient $\mu _{\mathrm {s}}$ and the cosine of the average scattering angle *g*. Typical values for *g* in enamel and dentin are 0.96 and 0.93^6^. Reduced albedo is given by $\alpha ^{'}=\frac{\mu ^{'}_{\mathrm {s}}}{\mu _ {{\rm tr}}}$.

The penetration coefficient $\mu _{\mathrm {eff}}= \sqrt{3 \mu _{\mathrm {a}} \mu _{\mathrm {tr}}}$ characterises the depth to which light typically penetrates tissue. Spatial frequency domain imaging uses the pattern penetration depth rather than simply the light penetration depth, consequently a modified pattern penetration coefficient is used $\mu ^{'}_{\mathrm {eff}} = \sqrt{\mu _{\mathrm {eff}}^{2} + (2 \pi f)^{2}}$, reflecting the depth to which the pattern typically penetrates the tissue. This allows the effective penetration coefficient to be varied independently of the transport coefficient using experimental parameters. The proportionality constant *A* is calculated from the effective reflection coefficient $R_{\mathrm {eff}}$ and accounts for the probability of photon flux exiting the tissue at the boundary. $R_{\mathrm {eff}}$ is calculated from the refractive index *n*.

5 $$\begin{aligned}&R_{\mathrm {eff}} \approx 0.668 + 0.0636n + \frac{0.710}{n} - \frac{1.440}{n^{2}} \end{aligned}$$

6 $$\begin{aligned}&A = \frac{1 - R_{\mathrm {eff}}}{2(1+R_{\mathrm {eff}})} \end{aligned}$$

Absorption is negligible in enamel at 850 nm^6^, allowing us to further simplify this solution to a function of reduced scattering coefficient $\mu ^{'}_{\mathrm {s}}$, the proportionality constant $A=0.102$ and the pattern frequency *f*:

7 $$\begin{aligned} I_{\mathrm {AC}} (f) = \frac{3 A B}{\Big (\frac{2 \pi f}{\mu ^{'}_{\mathrm {s}}} + 1\Big )\Big (\frac{2 \pi f}{\mu ^{'}_{\mathrm {s}}} + 3A\Big )} \end{aligned}$$

This expression allows the reduced scattering coefficient to be calculated for each pixel based on the AC image signal as a function of pattern frequency, shown in Fig. 1e. Fitting is performed with two free parameters, the reduced scattering coefficient $\mu ^{'}_{\mathrm {s}}$ and a dimensionless scaling term *B*. The use of a dimensionless scaling term accounts for factors such as illumination beam intensity and eliminates the need for reference measurements taken in phantoms.

It should be noted that whilst Eqs. (4) and (7) appear similar, $\mu ^{'}_{\mathrm {eff}}$ is a function of $\mu ^{'}_{\mathrm {s}}$, $\mu _{\mathrm {a}}$ and *f* and $\mu _{\mathrm {tr}}$ is a function of $\mu ^{'}_{\mathrm {s}}$ and $\mu _{\mathrm {a}}$. As a result, in addition to Eq. (4) having an additional variable to fit, care must be taken to ensure that optimal fit results for $\mu ^{'}_{\mathrm {eff}}$ and $\mu _{\mathrm {tr}}$ offer real, positive values of $\mu ^{'}_{\mathrm {s}}$ and $\mu _{\mathrm {a}}$ when including the effects of absorption. Consequently, fitting to Eq. (7) is both faster and more robust than fitting to Eq. (4), demonstrating the advantage of operating in a regime with negligible absorption such as that offered by the use of near infrared light.

This fit-based quantification approach is preferred to Monte Carlo techniques as this provides faster results, whilst also providing better precision and more insight into the fit quality than look-up table techniques. This fit quality allows us to identify when approximations in our model, such as that of homogeneous static tissue, break down. Fitting is performed with a nonlinear least-squares algorithm that returns a reduced scattering coefficient, a dimensionless scaling term and a quality of fit measurement $R^2$. The full process from demodulation to fit results is performed using custom MatLab code and is illustrated in Fig. 1c–f.

## Data Availability

Data used in this publication is available at 10.15128/r202870v89g.
